# Drug-Induced Liver Injury Secondary to Turmeric Supplement Containing Piperine: A Case Report

**DOI:** 10.7759/cureus.95076

**Published:** 2025-10-21

**Authors:** Nayeong Koo, Harthik Kambhampati, Michael Herman

**Affiliations:** 1 Osteopathic Medicine, Lake Erie College of Osteopathic Medicine, Bradenton, USA; 2 Gastroenterology, Borland Groover, Jacksonville, USA

**Keywords:** curcumin, dili, drug-induced liver injury, piperine, turmeric hepatotoxicity, turmeric-piperine interaction

## Abstract

We present the case of a 35-year-old male with jaundice, fatigue, pruritus, and decreased appetite, ultimately diagnosed with drug-induced liver injury (DILI) secondary to a turmeric (curcumin) supplement containing piperine. DILI is defined as liver injury caused by a drug, herbal product, or dietary supplement that results in biochemical or clinical evidence of liver dysfunction after exclusion of other causes. A comprehensive evaluation of this patient ruled out viral, autoimmune, ischemic, obstructive, and metabolic etiologies. Liver tests normalized following discontinuation of the supplement, and the patient experienced full clinical recovery. This case emphasizes the need for clinicians to recognize the potential hepatotoxicity of turmeric-piperine supplements and to evaluate supplement use in cases of unexplained liver injury.

## Introduction

DILI is the leading cause of acute liver failure in the United States and accounts for up to 20% of hepatotoxicity cases reported in large registries, including the Drug-Induced Liver Injury Network (DILIN) [1,2]. While prescription medications remain the most frequently implicated agents, herbal and dietary supplements (HDS) are increasingly recognized contributors to liver injury, as global use continues to rise [2].

Among these, turmeric (*Curcuma longa*), a widely used supplement known for its anti-inflammatory and antioxidant properties, has recently been reported as being hepatotoxic in certain individuals [3]. Curcumin, the principal bioactive component of turmeric, has limited oral bioavailability. To enhance systemic absorption, many commercial formulations include piperine, an alkaloid derived from black pepper. Piperine inhibits hepatic and intestinal glucuronidation and can increase curcumin’s bioavailability by up to 2,000% [4]. However, this potentiation may also elevate the risk of hepatotoxicity, as reported in several recent cases involving turmeric-piperine combinations [5,6].

We report a case of DILI in a 35-year-old male with hyperlipidemia on chronic statin therapy following ingestion of a turmeric supplement containing piperine. This case emphasizes the importance of clinician vigilance in assessing supplement use as part of the differential diagnosis of acute liver injury.

## Case presentation

A 35-year-old male with hyperlipidemia on statin therapy for three years presented to the emergency department with seven days of jaundice, fatigue, pruritus, and decreased appetite (Table 1). He denied abdominal pain, fever, nausea, vomiting, dark urine, or weight loss. He had no history of intravenous drug use, recent travel, blood transfusions, or high-risk sexual behavior. Alcohol consumption was minimal, with approximately one glass of scotch on several evenings per week, and he did not smoke or use illicit substances.

**Table 1:** Initial laboratory evaluation This table presents the patient’s initial laboratory values upon presentation, highlighting markers of liver function and other key diagnostic tests. ALT: Alanine aminotransferase, AST: Aspartate aminotransferase, ALP: Alkaline phosphatase, INR: International normalized ratio.

Test	Result	Reference Range
Total Bilirubin	10.0 mg/dL	0.2–1.2 mg/dL
ALT	2706 U/L	10–55 U/L
AST	1366 U/L	10–40 U/L
ALP	196 U/L	40–129 U/L
INR	1.1	0.8–1.2
Platelet Count	178 ×10⁹/L	150–400 ×10⁹/L
Acetaminophen Level	10 µg/mL	10-20 µg/mL

The patient reported starting a turmeric supplement containing curcumin and piperine six weeks prior to symptom onset and denied any additional turmeric or black pepper intake from dietary sources. Notably, several household members had recently experienced a self-limited gastrointestinal illness. He was admitted for monitoring and further workup (Table 2), and both the statin and turmeric supplement were discontinued. Liver enzymes demonstrated consistent improvement during hospitalization (Table 3). At six weeks post-discharge, the patient was asymptomatic, and liver function tests had fully normalized. He was counseled to avoid all dietary supplements unless specifically recommended by a healthcare provider.

**Table 2:** Further diagnostic workup This table summarizes the comprehensive diagnostic workup performed to exclude alternative etiologies of liver injury, including viral, autoimmune, metabolic, hemolytic, and biliary causes. ANA: Antinuclear antibody, CK: Antinuclear antibody, LDH:  Lactate dehydrogenase, MRCP: Magnetic resonance cholangiopancreatography.

Test	Result	Interpretation
Hepatitis A, B, C, E serologies	Negative	No viral hepatitis
ANA	Negative	No autoimmune hepatitis
CK	Normal	No muscle breakdown
Ceruloplasmin	26 mg/dL	Normal
Haptoglobin	Normal	No hemolysis
LDH	Normal	No hemolysis
Peripheral smear	Normal	No schistocytes or hemolysis
MRCP	Normal	No biliary obstruction

**Table 3:** Liver enzyme and bilirubin trend over time This table illustrates the chronological trend of the patient’s liver function markers following discontinuation of the turmeric supplement, demonstrating progressive biochemical improvement correlating with withdrawal of the suspected causative agent.

Timepoint	Total Bilirubin (mg/dL)	ALT (U/L)	AST (U/L)	ALP (U/L)
Day 1 (Admission)	10	2706	1366	196
Day 3	8.6	1921	996	—
Day 5	7.8	1782	888	—
Day 7 (Discharge)	4.2	1308	596	149
Six-Week Follow-Up	0.8	28	26	85

## Discussion

This case illustrates turmeric-associated DILI, which resolved following discontinuation of the supplement. The diagnosis was supported by a clear temporal relationship between supplement initiation and symptom onset, exclusion of alternative etiologies, and complete recovery upon withdrawal. According to Hy’s law, the concurrence of marked hepatocellular injury (alanine aminotransferase or aspartate aminotransferase (ALT or AST) >3× the upper limit of normal (ULN)) and hyperbilirubinemia (bilirubin >2× ULN) in the absence of significant cholestasis (alkaline phosphatase (ALP) <2× ULN) is predictive of severe DILI [7]. The patient’s laboratory profile fulfilled these criteria, underscoring the potential severity of turmeric-associated hepatotoxicity. The temporal pattern of injury strongly correlated with initiation of the turmeric-piperine supplement, and the patient’s prompt clinical and biochemical improvement following its discontinuation further supports causality. In contrast, the patient had demonstrated prolonged prior tolerance to statin therapy over several years without hepatic abnormalities, making statin-induced hepatotoxicity less likely.

Given the recent gastrointestinal illness among household members, infectious causes that can present with hepatotoxicity were considered. The patient underwent serologic testing for hepatitis A, B, C, and E, including Hepatitis B surface antigen (HBsAg), anti-HBs, anti-HBc, and anti-Hepatitis C virus (HCV) antibodies, all of which were negative. Testing for severe acute respiratory syndrome coronavirus 2 (SARS-CoV-2) and Adenovirus was not performed, which are infectious agents that can cause both hepatic and gastrointestinal manifestations. A blood ethanol level was also not measured, though the patient reported minimal alcohol intake.

Although turmeric is generally considered safe, reports of hepatotoxicity have been increasing, especially when combined with piperine [5]. Several mechanisms have been proposed to explain this hepatotoxicity. For example, the case series from DILIN suggests an immune-mediated mechanism, as piperine exposure and the HLA-B*35:01 allele were noted in some patients [6]. Other proposed mechanisms include idiosyncratic non-immune hepatotoxicity and oxidative stress [5,8-9]. Piperine may raise systemic curcumin levels by acting as an inhibitor of cytochrome enzymes, allowing reactive metabolites to accumulate [8]. High or sustained concentrations of curcumin can shift its activity from antioxidant to pro-oxidant, generating reactive oxygen species (ROS) that induce mitochondrial dysfunction and hepatocyte apoptosis [9]. This effect underscores the fine balance between curcumin’s protective and potentially harmful effects. Together, these pharmacokinetic and biochemical factors suggest that piperine and high-dose curcumin can synergistically increase susceptibility to liver injury. In this patient, HLA-B*35:01 testing was not performed, so a potential genetic predisposition cannot be excluded.

Beyond the complex mechanistic pathways, the causality assessment of turmeric-associated DILI is further complicated by the frequent presence of other hepatotoxic substances in commercial products. For instance, investigations have found lead chromate in certain turmeric products, which contribute to systemic and hepatotoxic risks [10,11]. The additive or synergistic effects of the contaminant with curcumin may exacerbate liver injury and hinder a clear diagnosis of turmeric as the sole cause. Clinically, DILI from turmeric-piperine often resembles acute viral hepatitis, with symptoms like significantly elevated aminotransferases, hyperbilirubinemia, fatigue, and pruritus [5]. The latency period typically ranges from one to four months [12]. Re-exposure to the supplement, whether accidental or intentional, often leads to a recurrence of the injury, sometimes with greater severity [12], which highlights the critical importance of counseling patients on complete avoidance of the product.

Looking at broader epidemiologic trends, the rising significance of supplement-induced hepatotoxicity is clear. HDS accounts for over 20% of reported DILI cases in the United States [2,13]. The U.S. Food and Drug Administration’s Center for Food Safety and Applied Nutrition (CFSAN) Adverse Event Reporting System (CAERS) indicates that liver injury is a commonly reported adverse effect, although the true incidence may be underestimated due to underreporting [14]. From a regulatory perspective, dietary supplements in the U.S. are marketed under the Dietary Supplement Health and Education Act (DSHEA) of 1994, which does not require pre-market safety testing [15]. This lack of oversight contributes to the perception of safety despite emerging evidence of toxicity. Thus, clinicians should actively screen for supplement use during evaluation of unexplained liver enzyme elevations. For future research, efforts should focus on clarifying dose-response relationships and further defining genetic predispositions [16]. Strengthened surveillance will be essential for better characterization and prevention of turmeric-piperine hepatotoxicity.

## Conclusions

This case highlights the importance of considering over-the-counter HDS, particularly those containing bioavailability enhancers like piperine, in the differential diagnosis of DILI. The definitive temporal relationship between supplement use and symptom onset, along with complete clinical and biochemical resolution upon discontinuation, supports the diagnosis. Clinicians should maintain a high level of suspicion for supplement-induced hepatotoxicity, especially when initial evaluations for more common causes of liver injury are inconclusive.

This report also emphasizes broader implications for public health and clinical practice. The growing recognition of supplement-related DILI, combined with a regulatory framework that lacks pre-market safety testing, underscores the need for improved patient education, enhanced surveillance, and regulatory oversight. Given the likely underreporting of turmeric-associated hepatotoxicity, further research is needed to clarify incidence, dose-response relationships, and underlying mechanisms, which will inform evidence-based recommendations and help ensure patient safety.
